# Generation of a Recombinant Gag Virus-Like-Particle Panel for the Evaluation of p24 Antigen Detection by Diagnostic HIV Tests

**DOI:** 10.1371/journal.pone.0111552

**Published:** 2014-10-24

**Authors:** Beatrice N. Vetter, Vanessa Orlowski, Katrien Fransen, Christoph Niederhauser, Vincent Aubert, Marcel Brandenberger, Diana Ciardo, Günter Dollenmaier, Thomas Klimkait, Stephan Regenass, Patrick Schmid, Volkmar Schottstedt, Franziska Suter-Riniker, Sabine Yerly, Cyril Shah, Jürg Böni, Jörg Schüpbach

**Affiliations:** 1 Swiss National Center for Retroviruses (SNCR), Institute of Medical Virology, University of Zürich, Zürich, Switzerland; 2 Institute of Tropical Medicine (ITG), Clinical Science, Antwerp, Belgium; 3 Blood Transfusion Service, Swiss Red Cross Berne (BSDSRK), Berne, Switzerland; 4 University Hospital, Service of Immunology and Allergy, CHUV, Lausanne, Switzerland; 5 Labor Synlab Luzern, Lucerne, Switzerland; 6 Viollier AG, Allschwil, Switzerland; 7 Center for Laboratory Medicine (ZLMSG), St. Gallen, Switzerland; 8 Department Biomedicine, Haus Petersplatz, University of Basel, Basel, Switzerland; 9 University Hospital, Clinic for Immunology, Zurich, Switzerland; 10 Department of Infectious Diseases, Cantonal Hospital St. Gallen (KSSG), St. Gallen, Switzerland; 11 German Red Cross Blood Transfusion Service West (DRK), Hagen, Germany; 12 University of Berne, Institute of Infectious Diseases, Berne, Switzerland; 13 University Hospitals (HUG), Laboratory of Virology, Genève, Switzerland; Institut National de la Santé et de la Recherche Médicale, France

## Abstract

**Background:**

Detection of HIV-1 p24 antigen permits early identification of primary HIV infection and timely intervention to limit further spread of the infection. Principally, HIV screening should equally detect all viral variants, but reagents for a standardised test evaluation are limited. Therefore, we aimed to create an inexhaustible panel of diverse HIV-1 p24 antigens.

**Methods:**

We generated a panel of 43 recombinantly expressed virus-like particles (VLPs), containing the structural Gag proteins of HIV-1 subtypes A-H and circulating recombinant forms (CRF) CRF01_AE, CRF02_AG, CRF12_BF, CRF20_BG and group O. Eleven 4^th^ generation antigen/antibody tests and five antigen-only tests were evaluated for their ability to detect VLPs diluted in human plasma to p24 concentrations equivalent to 50, 10 and 2 IU/ml of the WHO p24 standard. Three tests were also evaluated for their ability to detect p24 after heat-denaturation for immune-complex disruption, a pre-requisite for ultrasensitive p24 detection.

**Results:**

Our VLP panel exhibited an average intra-clade p24 diversity of 6.7%. Among the 4^th^ generation tests, the Abbott Architect and Siemens Enzygnost Integral 4 had the highest sensitivity of 97.7% and 93%, respectively. Alere Determine Combo and BioRad Access were least sensitive with 10.1% and 40.3%, respectively. Antigen-only tests were slightly more sensitive than combination tests. Almost all tests detected the WHO HIV-1 p24 standard at a concentration of 2 IU/ml, but their ability to detect this input for different subtypes varied greatly. Heat-treatment lowered overall detectability of HIV-1 p24 in two of the three tests, but only few VLPs had a more than 3-fold loss in p24 detection.

**Conclusions:**

The HIV-1 Gag subtype panel has a broad diversity and proved useful for a standardised evaluation of the detection limit and breadth of subtype detection of p24 antigen-detecting tests. Several tests exhibited problems, particularly with non-B subtypes.

## Introduction

Early diagnosis of HIV infection by timely HIV screening is one of the cornerstones of prevention of secondary transmission and an opportunity to initiate potentially beneficial, early antiretroviral treatment [1], [2]. Early diagnosis is important, as a large proportion of transmissions occur in the early phase of infection, due to the high viral load at this stage and an individual's unawareness of the infection [3]–[5]. The first viral markers detectable in patient plasma are viral RNA and p24 protein at a median of 9 and 16 days post infection, respectively [6], [7]. Antibodies to viral components are on average only detectable from 22 days post infection onwards [8]. The most economical way to diagnose early infection is by p24 antigen; screening tests that detect both antibodies and p24 antigen, so called 4^th^ generation or combination screening tests, were introduced into routine testing more than 15 years ago in Europe [9] and, more recently, also in the USA [10]. These tests have led to an increase in the identification of early HIV infections, attributed to the detection of p24 [9], [11], [12].

The high genetic diversity of HIV is a major challenge for any diagnostic test. HIV-1 consists of four phylogenetically different groups, M (major), O (outlier), N (non-M-non-O) and P. Group M viruses have been further divided into 9 different subtypes (A, B, C, D, F, G, H, J, K) and to date 55 circulating recombinant forms (CRFs) [13], some of which contribute substantially to the pandemic (such as CRF01_AE and CRF02_AG). The overwhelming majority of all HIV-1 infected individuals harbour viruses belonging to group M, but the global distribution of group M subtypes varies strongly [14]. The most prevalent subtype C largely circulates in sub-Saharan Africa and India, subtype A mostly circulates in Eastern Europe and Central Asia and subtype B mostly in Europe, the Americas and Oceania. The recombinant forms CRF01_AE and CRF02_AG are frequently found in Southeast Asia and West Africa, respectively. However, due to global mobility clades are not strictly confined to specific regions of the world.

Unlike for HIV nucleic acid tests, where a standardised and centrally distributed subtype reference panel was introduced several years ago [15], standardized reagents for assessing the quality of HIV-1 antigen detection in diagnostic tests are scarce. The only available reference reagent is a World Health Organization (WHO) standard, which consists of a single p24 antigen preparation from detergent-treated HIV-1, probably of subtype B [16]. Currently, subtype-sensitivity for antigen is established using seroconversion panels or culture-produced viruses. Seroconversion panels are expensive, in quantity limited, with unknown concentration of p24 antigen and limited subtype diversity. Culture-produced virus requires level-3 biosafety facilities for production, and standardisation based on HIV-1 RNA quantification may be impaired by subtype diversity [17].

In a novel approach to create a standardised, diverse and easy to produce panel of reagents, we have generated recombinantly expressed virus-like-particles (VLPs), expressing the Gag proteins from various subtypes in a subtype B backbone. These VLPs are non-infectious, produced by transfection under low-level biosafety conditions and easily standardised based on their content of a uniform, backbone-derived reverse transcriptase. Therefore, they represent an inexhaustible source for a standardised evaluation of p24 antigen tests, which covers the entire breadth of HIV-1 diversity. Here we used this panel to evaluate commercial diagnostic HIV tests.

## Materials and Methods

### Ethics statement

All HIV-1 RNA samples used in this study were obtained from residual material after routine genetic resistance testing. No informed consent was obtained, because the study used only the viral sequences but no patient information.

### Viruses and cloning procedure

Viral RNA isolates used for *gag* cloning were chosen based on the HIV-1 subtype determined by sequencing the *protease-reverse transcriptase (pr-rt)* region of *pol* for drug resistance testing. All primers used in the cloning procedure are listed in table S1 in file S1. Due to the overlapping *gag-protease (gag-pr)* reading frame in the viral genome, the entire *gag-pr* part of the viral genome was cloned into the second generation lentiviral vector pCMVΔ8.91 [18] by replacing the excised *gag-pr* with the isolate-derived *gag-pr* part via unique restriction sites. To this end a unique MluI restriction site was introduced in the vector at the 5′ end (position 844–849) immediately upstream of the start of the *gag*-coding region, and a PvuI restriction site was introduced as a silent mutation at the 3′ end (position 2625–2630) of the *pr*-coding region. A pre-existing PvuI restriction site in the *amp*-coding region of the plasmid (position 8466–8471) was deleted by silent mutation. All mutations were performed using the XL-site-directed mutagenesis kit (200521) from Stratagene. cDNA synthesis from isolate RNA was performed at 42°C for 60 min followed by 2 min at 96°C, using the PrimeScript One-Step RT-PCR ver. 2 (RR055B) from TaKaRa. The *gag-pr* part of the viral genome was either amplified directly from cDNA or via nested PCR after an intermediate first-round PCR step. For direct *gag-pr* amplification or intermediate first-round PCR, 250 nM of forward and reverse primers were added directly to the synthesised cDNA, and PCR was performed according to cycling parameters in table S2 in file S1. One µl of the first round PCR product was used in a subsequent nested *gag-pr* amplification PCR, using Phire Hot Start DNA Polymerase (Life Technologies). *Gag-pr* PCR products and the modified backbone pCMVΔ8.91 were purified, digested and ligated according to standard molecular biology procedures. Ligated plasmids were transformed into z-competent (T3001, Zymogen) DH5alpha E.coli bacteria. Mutagenesis and insertion of *gag-pr* into the vector were verified by sequencing. The recombinant g*ag-pr* nucleotide sequences were submitted to the National Center for Biotechnology Information (NCBI) GenBank database and assigned accession numbers are listed in table S3 in file S1.

### VLP production, purification and quantification

For the production of VLPs, 40 µg plasmid were transfected over night into 293T-cells, plated at 2×10^6^ cells/75 cm^2^ flask, using polyethylenimine transfection reagent at twice the amount of transfected DNA. Supernatants were collected 24 h and 48 h after medium change and stored at 4°C. For VLP purification, pooled supernatants were filtered (0.2 µm), and pelleted through a 32% sucrose cushion in a Sorvall WX Ultra 100 centrifuge (Thermo Scientific), swing-out rotor AH-629/36, for 2 h and 141′000 g at 4°C. The pellet was resuspended in 1 ml cold PBS, and 50–100 µl aliquots were stored at −80°C. Reverse-transcriptase (RT) activity of two dilutions of VLP preparations (1∶4 and 1∶8) was measured in duplicates using the Roche Colorimetric Reverse Transcriptase Assay (article 11468120910), and RT-activity was quantified based on the kit's recombinant RT-standard (expressed quantitatively as ng/ml).

### VLP *gag* subtyping and phylogenetic analysis

The subtype of cloned *gag* was verified using the NCBI genotyping tool (http://www.ncbi.nlm.nih.gov/projects/genotyping/formpage.cgi) and the 2009 reference set. In cases where the assigned subtype in *gag* differed from the originally assigned *pol-*subtype, the *gag*-subtype was chosen for denomination. In case of typing difficulties due to mixed genotypic subtyping results, the subtype predominant in p24 was chosen. P24 amino acid pairwise sequence alignments were performed using Clustal Omega (DNASTAR Lasergene MegAlign Pro v11.2.1), and the Gag phylogenetic tree was constructed by the neighbour joining method (Clustal W, DNASTAR Lasergene MegAlign v11.2.1) and visualized using FigTree v1.3.1.

### Panel preparation and standardisation for diagnostic test evaluation

All VLP preparations used in diagnostic tests were diluted in pooled HIV-negative human plasma (Swiss Red Cross Blood Donation Centre Zurich, Schlieren). Dilutions were prepared in a single batch per VLP to ensure comparable source material. Batches were aliquoted and frozen. The VLP input for diagnostic test evaluation was based on the WHO p24 international standard (NIBSC 90/636, expressed in International Units [IU]) as follows: using the bioMérieux VIDAS HIV p24 II quantitative antigen test, five concentrations of the WHO p24 standard (20, 10, 4, 1, 0.8 IU/ml, diluted in negative plasma) were quantified for p24 pg/ml content (table S4 in file S1) and an average conversion rate was derived. To estimate the relationship between RT-quantity and p24 pg/ml, two concentrations of RT (0.025 and 0.008 ng/ml) of four subtype B VLPs (pBV6, 8, 11, 15) were quantified for p24 pg/ml on the bioMérieux VIDAS HIV p24 II (figure S1), and the ratio of the average p24 pg/ml values to input RT ng/ml quantity was derived. We thus determined that a VLP input of 0.001 ng/ml RT was approximately equivalent to 10.3 pg/ml p24 and 2 IU/ml WHO p24 standard. For the evaluation of diagnostic tests, three dilutions of each VLP were prepared with RT-quantities corresponding to 50, 10 and 2 IU/ml WHO p24 standard (i.e. 0.025, 0.005 and 0.001 ng/ml RT, respectively). Frozen aliquots were stored at −20°C until analysis.

### Evaluation of diagnostic HIV tests

All commercial diagnostic HIV tests included in this study, analytical platforms and laboratories performing the tests are listed in table S5 in file S1. Sample aliquots were shipped frozen on dry ice. Sample analysis was performed in a blinded fashion, and two aliquots of HIV-negative human plasma without VLPs were included for each test. Samples were treated according to each participating laboratory's approved pre-analytical process for the respective test to be conducted.

### Heat-denaturation

Heat denaturation was performed according to published methodology [19] in order to see how this procedure for immune complex disruption and elimination of antibody-mediated interference in antigen tests affected the detection of p24 antigen. Briefly, using the 50 IU/ml VLP samples, 100 µl were treated with 50 µl 3× virus disruption buffer for 10 minutes at room temperature. Samples were subsequently diluted to 10 IU/ml by adding 350 µl PBS and boiled at 100°C for 5 min in a pre-heated dry heat block.

## Results

### VLP panel characteristics

Our aim was to create a VLP panel representing the wide variety of HIV-1 sequences in the *gag* region. By replacing *gag* of the vector-encoded viral genome with the respective sequences from different clades of HIV-1, we generated VLPs which contained all the structural proteins from the clades of our choice, while the RT of the particles originated from the subtype B backbone plasmid. The enzyme gene was thus conserved in all constructs, enabling sequence-independent quantification of all VLPs with a commercial RT test. The resulting panel consisted of 42 VLPs with isolate-derived *gag* of HIV-1 subtypes A, B, C, D, F, G, H, CRF01_AE, CRF02_AG, CRF12_BF, CRF20_BG, and group O. Additionally we included the molecular clone NL4–3 [20] in pCMVΔ8.91 (subtype B), resulting in a panel of 43 VLPs.

**Figure 1:**
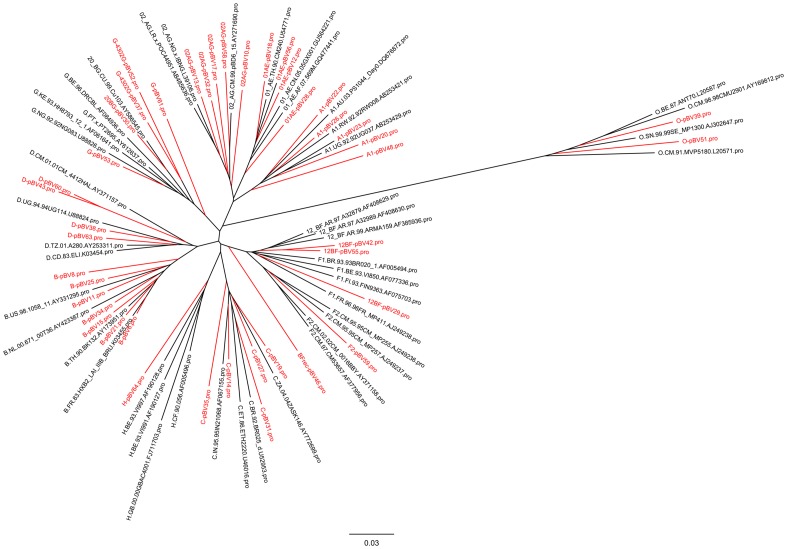
Phylogenetic relationship between Gag amino acid sequences of VLP panel members (red) and Los Alamos National Laboratory (LANL) subtype reference sequences (black). Gag subtype reference sequences were downloaded from the LANL website http://www.hiv.lanl.gov/content/sequence/NEWALIGN/align.html and filtered for subtypes present in the VLP panel. The phylogenetic tree was constructed using the neighbour joining method (Clustal W). The scale bar indicates branch length, expressed as the number of substitutions per site.

Subtyping of the *gag* region of the 42 isolate-derived VLP constructs showed good agreement with the originally assigned subtype in *pr-rt* in all but seven cases. In three of these, the *gag* subtype clearly differed from *pr-rt*, with the following discrepancies for *gag/pr-rt*: A1/D (pBV48); CRF02_AG/F2 (pBV58); CRF02_AG/A1 (pBV10). Evaluation of the original *pr-rt* sequences generated during drug resistance testing showed concordance of the *rt* region with the originally assigned subtype. Hence, for these isolates there was a true subtype discrepancy between *gag* and *pol*, and the subtype for our panel was taken according to *gag*. One VLP had a subtype assignment change from CRF14_BG to CRF20_BG as a result of using the updated NCBI reference sequence set of 2009 which contains a larger number of CRFs (the reference set of 2005 had been used for the original *pr-rt* subtype). Subtyping of three VLP *gags* originally assigned F1 was most difficult (pBV42, 46 and 55). They aligned to a mixture of F1 and CRF12_BF as well as other recombinant reference sequences across *gag*. Phylogenetic analysis of their amino acids sequences (figure 1) showed clustering of two of these VLPs more closely with CRF12_BF reference sequences (pBV42 and pBV55), whereas the third VLP (pBV46) could not be assigned a specific CRF. The phylogenetic tree also showed that pBV29 clustered together with the F1 subtype reference sequences although the original as well as our genotyping analysis had identified this viral isolate as CRF12_BF. We thus decided to keep this subtype assignment. All other VLP Gag amino acid sequences clustered into the selected clades and together with the respective Los Alamos National Laboratory (LANL) subtype reference sequences. This confirmed our subtype assignment, also for those VLPs in which *gag* and *pr-rt* subtype assignment differed.

To assess the diversity of our panel, we conducted pairwise amino acid sequence comparison of p24 proteins within each clade that had more than one representative member (table 1). The same was done with matched clades for p24 amino acid sequences of the LANL reference subtypes. Overall weighted average diversity of our panel was 6.7% (±1.2) and 5.1% (±1.8) for the LANL reference sequences. Within clades, our panel mainly showed similar amino acid diversity to the LANL subtype references. The higher diversity of our panel in some clades may result from less clean subtypes in our virus isolates compared to the reference sequences.

**Table 1:** Amino acid sequence divergence (%) of p24 in VLP panel and LANL reference subtypes after pairwise sequence alignment.

	VLP panel		LANL reference subtypes	
	n	p24	n	p24
A1	5	8.0±1.9	3	8.3±1.6
B	7	5.4±1.6	4	3.3±1.9
C	5	6.1±0.9	4	5.2±1.8
D	4	7.6±3.9	4	4.1±1.2
CRF12_BF	4	6.5±2.3	3	3.6±0.3
F2	1	N/A	N/A	N/A
G	4	6.7±1.8	4	4.6±2.8
CRF20_BG	1	N/A	N/A	N/A
H	1	N/A	N/A	N/A
CRF01_AE	4	4.7±1.8	3	4.1±1.3
CRF02_AG	5	8.3±1.9	3	4.3±0.9
group O	2	8.2	4	8.3±1.3
**weighted average**		**6.7±1.2**		**5.1±1.8**

N/A  =  not applicable; n =  number of sequences.

### Evaluation of diagnostic tests

#### Diagnostic sensitivity

**Figure 2:**
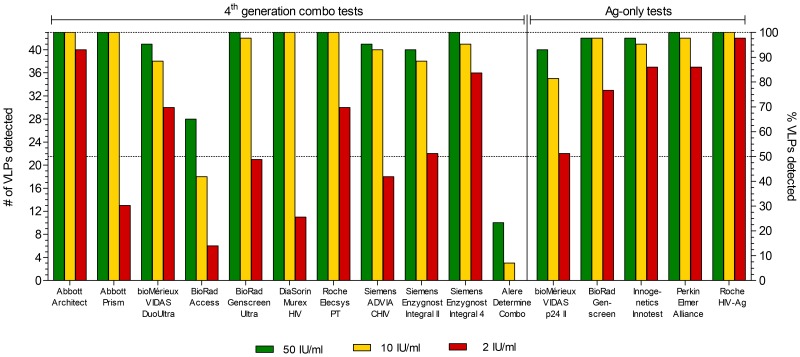
Total number of VLPs detected per input concentration for each 4^th^ generation combo test and Ag-only test. Three input concentrations (see legend) of VLPs diluted in negative human plasma were analysed blindly and VLPs were scored “detected” as per the manufacturers instruction for unequivocally positive samples. The left *y*-axis shows numbers of VLPs detected while the right *y*-axis shows the corresponding percentage. Dotted lines indicate 50% and 100% detection.

In total, 16 diagnostic tests were evaluated, 11 of which were 4^th^ generation combo tests and five were antigen-only tests (for details see table S5 in file S1). For each VLP, three input concentrations were prepared in negative human plasma. For the lowest input concentration, an RT-activity corresponding to a p24 concentration of 10.3 pg/ml or 2 IU/ml of the WHO p24 standard was chosen (see methods). This concentration reflects the minimal requirement for p24 antigen detection in order to obtain CE-marking, the European equivalent of the U.S. FDA approval [21]. The intermediate and highest input contained 10 IU/ml and 50 IU/ml, respectively, corresponding to about 50 and 250 pg/ml p24. Dilutions of the WHO standard at these concentrations were also included in the panel. VLPs were scored “detected” as per the manufacturers instruction for unequivocally positive samples, i.e. samples in a defined greyzone were scored as “not detected”. Figure 2 shows the total number of VLPs detected per input concentration for each test. Of the 11 fourth generation tests, the Abbott Architect, Roche Elecsys PT and Siemens Enzygnost Integral 4 showed excellent subtype breadth and high sensitivity, meaning they detected all 43 VLPs at the 50 IU/ml input concentration and the majority of VLPs at the 10 and 2 IU/ml concentration. The BioRad Genscreen Ultra, Abbott Prism and DiaSorin Murex HIV also showed high subtype breadth at the two highest input concentrations but reduced sensitivity at the 2 IU/ml concentration. The bioMérieux VIDAS DuoUltra, Siemens Enzygnost Integral II and Siemens ADVIA CHIV did not detect 100% of VLPs at any concentration. However, certainly the bioMérieux VIDAS DuoUltra had overall good sensitivity for the VLPs that were detectable. Tests with extremely poor sensitivity and low subtype breadth were the BioRad Access and Alere Determine Combo. They clearly failed to detect the majority of VLPs.

For the antigen-only tests, sensitivity was generally higher compared to the 4^th^ generation combo tests. The Roche HIV-Ag and Perkin Elmer Alliance (a test neither CE-marked nor FDA approved) had excellent subtype breadth and high sensitivity, detecting the large majority of VLPs even at the 2 IU/ml concentration. The BioRad Genscreen and Innogenetics Innotest failed to detect all VLPs, but still exhibited good sensitivity at low input concentration. Only the bioMérieux VIDAS p24 II showed lower subtype breadth, combined with comparably low sensitivity for this test category. The lower sensitivity could be in part due to the relatively high clear-positive 5 pg/ml cut-off. If the equivocal 3 pg/ml cut-off was applied, subtype breadth was still below 100% but sensitivity at the 2 IU/ml input increased to 65% (data not shown).

**Figure 3:**
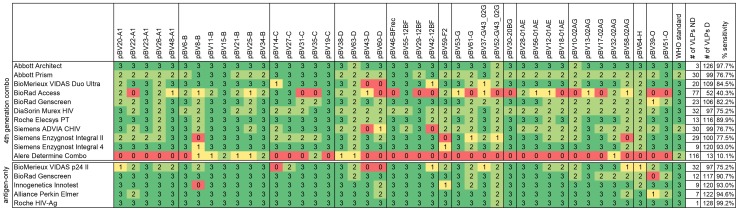
Overview of all results per test and VLP. Numbers indicate how many input concentrations were detected per VLP and test, i.e. 3 = 50, 10 and 2 IU/ml concentrations detected, 2 = 50 and 10 IU/ml detected, 1 = 50 IU/ml detected, 0 =  VLP not detected at any concentration. The overall sensitivity for each test was calculated as number (#) of VLPs detected (D)/total number of VLPs (n = 129, i.e. # of VLPs detected + # of VLPs not detected [ND], excluding the WHO p24 standard).

Figure 3 details the result of each test for individual VLPs and the WHO standard. Numbers in the table indicate how many input concentrations were detected per VLP and test. The resulting overall frequency of positive samples for each test (VLP samples detected/all 129 VLP samples) is listed at the right end of the table. Failure to detect a VLP was not restricted to non-B subtypes, however, certainly for the 4^th^ generation combo tests, subtype B VLPs were detected most efficiently. Compared to the 4^th^ generation tests, the group of antigen-only tests generally exhibited a higher sensitivity for some of the non-B clades. This is probably due to the higher technical sensitivity of antigen-only tests, which becomes most apparent when comparing the total numbers of VLPs detected by tests of the same manufacturer (such as BioRad Genscreen and the Roche tests). The WHO p24 reference standard was detected by all tests, however, at the 2 IU/ml concentration the Abbott Prism and Siemens ADVIA CHIV narrowly missed the cut-off (data not shown), and the Alere Determine Combo scored clearly negative.

#### Heat-treated VLPs

**Figure 4:**
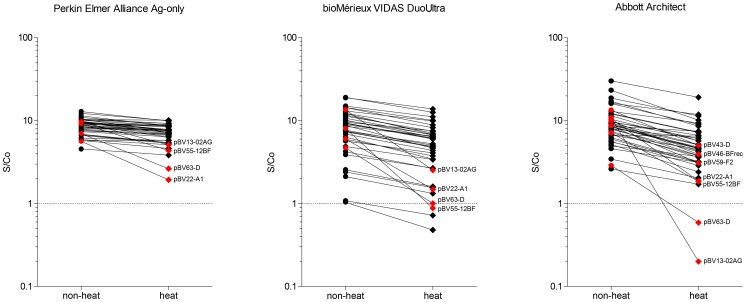
Detection of VLPs before and after heat-denaturation at 10 IU/ml. VLP preparations of 50 IU/ml were diluted at 1:5 in PBS and heat-denatured for 5 min at 100°C. Results for the non-heat treated VLPs for the Abbot Architect and Perkin Elmer Alliance were taken from the complete panel analysis and heat-treated VLP measurement was conducted separately. For the bioMérieux VIDAS DuoUltra, heat and non-heat treated samples were analysed in parallel. Highlighted in red are VLPs with loss of p24 detection between 1.9–3-fold for the Perkin Elmer Alliance and ≥3-fold for bioMérieux VIDAS DuoUltra and Abbott Architect.

A modification of the p24 antigen test involving heat denaturation of the serum or plasma sample prior to testing has been demonstrated to improve p24 detection considerably in samples also positive for HIV-specific antibodies, rendering p24 testing feasible for diagnosing paediatric HIV-1 infection and, perhaps even use it instead of HIV-1 RNA load for antiretroviral treatment monitoring [22]–[24]. We therefore also evaluated the effect of heat denaturation (100°C/5 min) on the detection of the VLPs for three different tests: the antigen-only Perkin Elmer Alliance and the 4^th^ generation combo tests bioMérieux VIDAS DuoUltra and Abbott Architect. The Perkin Elmer Alliance has been used extensively with heat-denatured samples [25] and the antigen-only test of bioMérieux (VIDAS p24 II) has also been used to this end in one study [26]. Figure 4 shows S/Co ratios measured in native compared to heat-treated VLP samples at an input concentration of 10 IU/ml (VLPs undetectable at 10 IU/ml in native samples were excluded, see figure 3). For the Perkin Elmer Alliance, no sample showed a ≥0.5 log (or 3-fold) drop in S/Co ratio after heat treatment. The greatest loss in signal was between 1.9–2.9 fold for four VLPs indicated in red in figure 4. The average drop in S/Co ratio for the remaining samples was 0.06 log. The S/Co ratio for the bioMérieux VIDAS DuoUltra dropped by ≥0.5 log for four VLPs, and the average drop for the remaining VLPs was 0.18 log. For the Abbott Architect, the S/Co ratio after heat-denaturation dropped by more than half a log (or 3-fold) for seven VLPs, and the average drop in S/Co ratio for the remaining VLPs was 0.23 log. Interestingly, detection of the same four VLPs was impaired after heat-treatment in all three tests, suggesting that at least one of the two antibodies employed in the p24 sandwich-detection used in these tests binds the same epitope.

## Discussion

In this study, we have created a panel of VLPs with the aim to represent the broad diversity of HIV-1 Gag proteins. We used this panel for evaluation of commercial tests for the detection of p24 antigen, including both the 4^th^ generation combo screening tests and dedicated antigen-only tests.

### Virus-like particles

In addition to *gag*, we decided to clone the *pr* part of the viral genome into a subtype B *pol* reverse-transcriptase-integrase background to avoid the generation of a hybrid PR from mixed subtypes due to the overlapping *gag-pol* reading frame (see Materials and Methods). We thus also ensured optimal conditions for efficient processing of Gag by its matching protease. The ability of PR to process Pol polyproteins from heterogeneic subtypes is not well studied, however many CRFs have subtype recombinations within Pol, suggesting a certain flexibility. Furthermore, a recent study on the conservation rate of PR cleavage sites in Gag-Pol found a high degree of conservation in Pol, suggesting that it was evolutionary not necessary to adapt Pol cleavage sites despite sequence variability in PR [27].

With the 43 viral isolates we achieved a good genetic diversity of the VLP panel. Comparison of the diversity of our panel with that of matched LANL subtype reference strains showed that the two diversities were similar, thus suggesting a good representation of global diversity in our panel. Notably, we found subtype differences in *gag* and *pr-rt*. Ideally, subtype identification should be performed across the whole genome, however in routine diagnostic genotyping of patient samples this is not the case, which should be kept in mind when drawing conclusions about subtype-sensitivities of any given diagnostic test. Clades not represented in our panel include J and K as well as members of HIV-1 groups N and P and HIV-2. They all have very low global prevalence and were not available to us. In case of HIV-2, the practical relevance of p24 detection might be low anyway, as the concentration of HIV-2 particles in plasma is usually too low for detection by p24 antigen tests [28]. Furthermore, only three cases of acute HIV-2 infection have been described since the first description of HIV-2 almost three decades ago, making this an extremely rare event [29]–[31]. Nevertheless, with the current composition of the panel we have already demonstrated significant differences in the performance of diagnostic tests, and due to the technical flexibility of the VLP DNA construct it will be easy to add more subtypes and CRFs as needed.

### Evaluation of diagnostic tests

The diagnostic HIV tests were evaluated at three p24 concentrations, starting with 2 IU/ml as the minimum sensitivity required for CE-marking, up to a very high p24 concentration of 50 IU/ml in order not to miss any VLPs which might be detected with lower sensitivity due to their subtype. In our hands, a concentration of 2 IU/ml of the WHO standard was equivalent to 10.3 pg/ml p24, which compared well with previously published estimates [32]. Standardisation of VLP input was based on particle-associated RT-activity, and normalisation was controlled by measuring p24 content of four RT-normalised subtype B VLPs (figure S1). Their p24 concentration compared well, though the p24 content of pBV8-B was somewhat lower, which might be a result of an amino acid point mutation of this VLP in a highly conserved region. Suboptimal detection of this VLP was also observed across a range of other tests (figure 3). Despite this limitation, the very comparable p24 content of the three other VLPs confirmed good input normalisation.

Among the 4th generation combo tests evaluated, the Abbott Architect had the highest frequency of positive samples, also detecting all tested subtypes. A comparable result (around 90% sensitivity and detection of all subtypes) was only achieved by two other tests of this category, namely the Roche Elecsys PT and Siemens Enzygnost Integral 4. Superior performance in breadth of subtype detection of the Abbott Architect and the Roche Elecsys PT was also observed in a range of other recent smaller comparative studies [33]–[36]. Published independent evaluations of the Siemens Enzygnost Integral 4 do not yet exist, as this test has only been available for a short time. Due to limited overlap in test combinations between our study and previously published evaluations, direct comparison of results of the other tests is not easy. The largest recent study compared 10 fourth generation screening tests, seven of which overlapped with our study [34]. The studies agreed on best-performing (Abbott Architect and Roche Elecsys PT) and worst-performing (BioRad Access) tests, but the ranking for the other tests varied. This is most likely due to the different make-up and concentrations of the test panels, highlighting the need for standardised test material in such evaluation studies.

In our study, overall sensitivity of the antigen-only tests was better for the majority of tests, even for those that did not detect all subtypes (BioRad Genscreen and Innogenetics Innotest). Among the best-performing antigen-only test was the Perkin Elmer Alliance, which is remarkable, considering that this test was developed more than 20 years ago when much less was known about subtype diversity.

Variability in analytical test sensitivity for the different HIV-1 subtypes was most apparent at the lowest input concentration of 2 IU/ml. Here, several tests which performed well at the two higher concentrations, showed a large drop in VLP detection (figure 2), indicating a relatively high limit of detection. This is worrisome, considering that the minimum sensitivity required (2 IU/ml or 10.3 pg/ml p24) already allows for a relatively high viral load: 10 pg/ml p24 equal approximately 100′000 copies/ml RNA (assuming 5′000 Gag molecules per viral particle [37]), a viral load also measured in a recent study correlating p24 antigen sensitivity of the Abbott Architect with RNA viral load [38]. Highly sensitive tests, such as the 4^th^ generation combo tests Abbott Architect and Siemens Enzygnost Integral 4 as well as the antigen-only tests, detected most of the 2 IU/ml VLP samples with signals clearly above the positive test cut-off. This is desirable to enable detection of primary infection as early as possible. In the manufacturer's manual, many commercial tests indicate an LOD well below 2 IU/ml when using subtype B reagents such as the WHO p24 antigen standard or the AFSSAPS panel (Agence Française de Sécurité Sanitaire des Produits de Santé [39]) (see table S5 in file S1). This, of course, shortens the time to p24 detection, however, as we and others show [34], not necessarily for all subtypes. To ensure a more appropriate determination of the LOD, equal analytical sensitivity for a range of different subtypes should be a regulatory requirement.

Considering the recently obtained FDA approval of the Alere Determine HIV-1/2 Ag/Ab Combo, we were surprised to see how poorly this test performed with our panel. Not only did it miss all but a few of the non-B VLPs, it also was the least sensitive test for the subtype B isolates. Our results explain why the test also performed poorly in several other studies using patient samples [40]–[42]. In combination, these results suggest that the Alere Determine Combo cannot replace a 4^th^ generation screening test performed in the laboratory.

### Detection of VLPs after heat treatment

Previous studies have shown that a short boiling of diluted plasma or serum samples effectively inactivates all interference by antibodies with the detection of p24 in an antigen test [43]. Complementation of the antigen test with a signal amplification procedure has further increased the diagnostic and analytical sensitivity, leading to the so-called “ultrasensitive p24 antigen test” (Up24) [44] recommended by the WHO as an alternative to PCR for diagnosing HIV infection of newborns in resource-poor settings [45]. Equally, during chronic infection, the majority of p24 in plasma is bound in immune-complexes and only becomes detectable after heat-mediated immune-complex disruption [46]. The use of this simple methodology has great potential in resource-limited settings, where PCR-based viral load monitoring is often not possible. The Perkin Elmer Alliance in combination with heat denaturation has been used successfully in several studies as inexpensive alternative to RNA [25], [47]. As it is fairly labour-intensive, it would be desirable to investigate the use of fully automated platforms for p24 detection in heat-denatured plasma samples. However, a pre-requisite is the preservation of the p24 antibody epitope under heat-treatment. Our evaluation of the VLP panel to this end showed that the majority of VLPs were still detectable, albeit with decreased S/Co ratios in case of the Abbott Architect and the bioMérieux VIDAS DuoUltra. The loss in signal for the Perkin Elmer Alliance Up24 may be less serious than it appears here, considering that this test has a decreased non-specific background for samples from uninfected controls [19], thus permitting to lower the cut-off of reactivity. If, by heat denaturation, the cut-off could likewise be lowered for the Abbott Architect and VIDAS test, they might become attractive alternatives to the Perkin Elmer Alliance Up24 for ultrasensitive p24 detection, as they offer fully automated testing. At present, the Perkin Elmer Alliance exhibited the highest signal/cut-off ratios, and there was no general decrease of p24 detection, except for the four VLPs which were clearly impacted by heat-denaturation in all three tests. It is unknown why these four isolates are so fundamentally different from the others.

## Conclusion

Here we demonstrate the usefulness of our recombinant VLP panel in assessing diagnostic tests for HIV-1 p24 antigen detection. We show that both the breadth of subtype-sensitivity as well as the analytical limit of detection can be assessed by this panel. The use of the VLPs' conserved RT enzyme activity for p24 input standardisation makes the panel easy to employ in future studies and by other laboratories, as any newly produced stock can simply be quantified by a commercially available RT test. We thus believe that the panel will prove valuable for the evaluation of diagnostic reagents in the future, because it is comparatively easy to produce and standardise and represents an inexhaustible non-infectious source of diverse HIV-1 p24 antigens.

## Supporting Information

Figure S1 **p24 quantification of four subtype B VLPs at defined RT ng/ml inputs.** VLPs were diluted to 0.025 ng/ml RT and 0.008 ng/ml RT in negative human plasma and p24 quantities were measured on the bioMérieux VIDAS p24 II.
(TIF)

File S1 **Tables S1–S5.** Table S1: Primers used in this study; Table S2: PCR cycling conditions; Table S3: NCBI GenBank accession numbers for *gag-pr* VLP nucleotide sequences; Table S4: p24 quantification of WHO standard on the bioMérieux VIDAS p24 II; Table S5: Commercial HIV antigen/antibody and antigen-only tests evaluated in this study.
(DOCX)
