# Supplementary figures and images for: Generation of a Recombinant Gag Virus-Like-Particle Panel for the Evaluation of p24 Antigen Detection by Diagnostic HIV Tests
Source: PLoS One. 2014 Oct 24;9(10):e111552. doi: 10.1371/journal.pone.0111552 (PMC4208835; doi:10.1371/journal.pone.0111552)

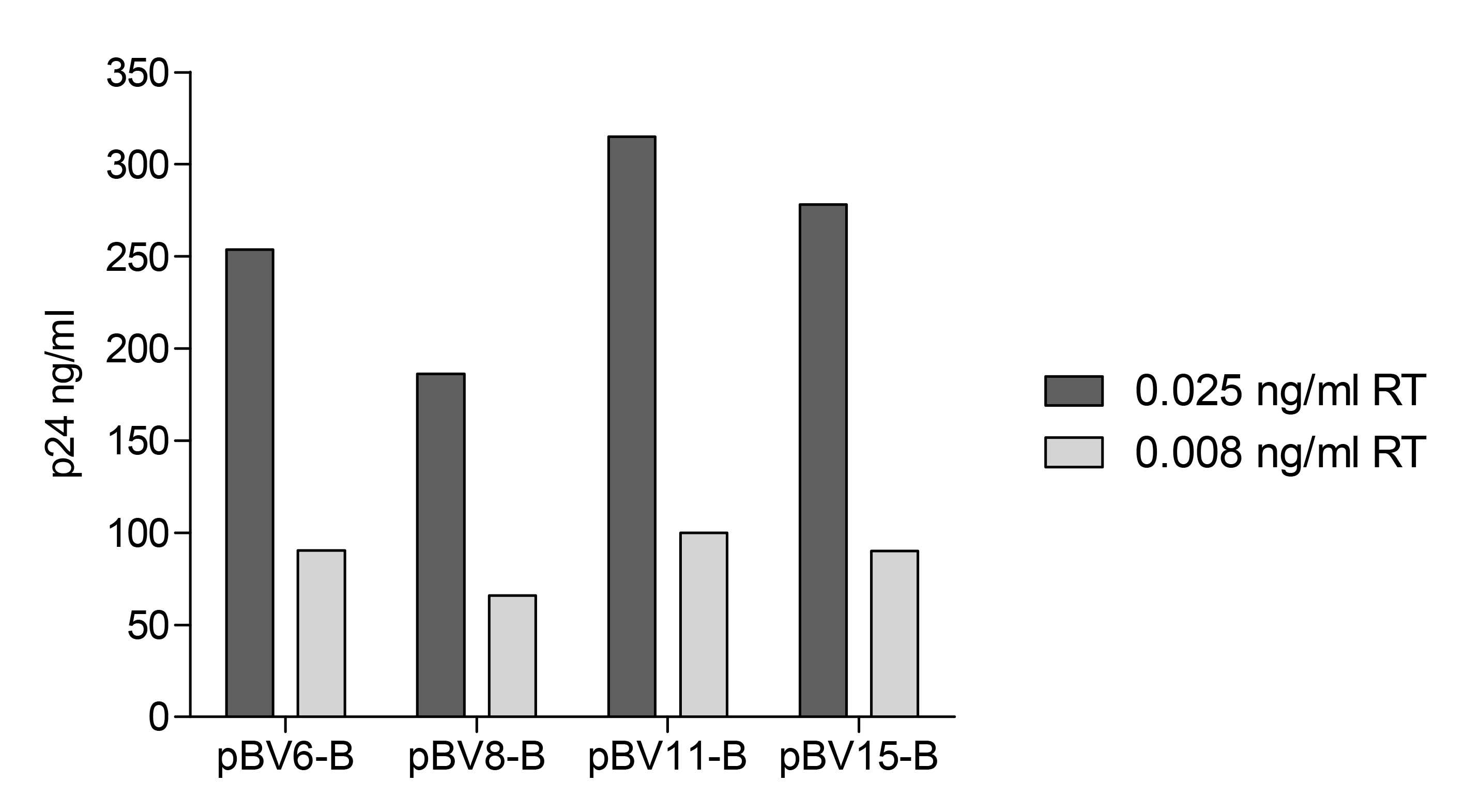

Supplement: Figure S1 — p24 quantification of four subtype B VLPs at defined RT ng/ml inputs. VLPs were diluted to 0.025 ng/ml RT and 0.008 ng/ml RT in negative human plasma and p24 quantities were measured on the bioMérieux VIDAS p24 II. (TIF) [file pone.0111552.s001.tif]
