# Supplementary material for: Generation of a Recombinant Gag Virus-Like-Particle Panel for the Evaluation of p24 Antigen Detection by Diagnostic HIV Tests
Source: PLoS One. 2014 Oct 24;9(10):e111552. doi: 10.1371/journal.pone.0111552 (PMC4208835; doi:10.1371/journal.pone.0111552)
Supplement: File S1 — Tables S1–S5. Table S1: Primers used in this study; Table S2: PCR cycling conditions; Table S3: NCBI GenBank accession numbers for gag-pr VLP nucleotide sequences; Table S4: p24 quantification of WHO standard on the bioMérieux VIDAS p24 II; Table S5: Commercial HIV antigen/antibody and antigen-only tests evaluated in this study. (DOCX) [file pone.0111552.s002.docx]

| Table S1: Primers used in this study | |  |  |  |  |  |
| --- | --- | --- | --- | --- | --- | --- |
| **name** | **description** | **use** | **target** | **position** | **sense** | **sequence 5'-3'** |
| OBV11 | site-directed-mutagenesis | introduction of MluI restriction site | pCMVΔ8.91 | 833-860* | forward | GAGGGGCGGCGACGCGTGAGAGATGGGT |
| OBV12 | site-directed-mutagenesis | introduction of MluI restriction site | pCMVΔ8.91 | 833-860* | reverse | ACCCATCTCTCACGCGTCGCCGCCCCTC |
| OBV13 | site-directed-mutagenesis | elimination of PvuI restriction site | pCMVΔ8.91 | 8451-8483* | forward | ACTTACTTCTGACAACCATCGGAGGACCGAAGG |
| OBV14 | site-directed-mutagenesis | elimination of PvuI restriction site | pCMVΔ8.91 | 8451-8483* | reverse | CCTTCGGTCCTCCGATGGTTGTCAGAAGTAAGT |
| OBV15 | site-directed-mutagenesis | introduction of MluI restriction site | pCMVΔ8.91 | 2601-2654* | forward | GCACTTTAAATTTTCCCATTAGTCCGATCGAGACTGTACCAGTAAAATTAAAGC |
| OBV16 | site-directed-mutagenesis | introduction of MluI restriction site | pCMVΔ8.91 | 2601-2654* | reverse | GCTTTAATTTTACTGGTACAGTCTCGATCGGACTAATGGGAAAATTTAAAGTGC |
| OBV20 | cDNA synthesis all subtypes | all subtypes | viral RNA | 2532-2549 | reverse | AAAATTTAAAGTGCAGCC |
| OBV27 | cDNA synthesis subtypes B, D, F1 | subtypes B, D, F1 | viral RNA | 2532-2549 | reverse | AAAATTTAAAGTGCAACC |
| OBV28 | cDNA synthesis subtypes G, CRF02_AG | subtypes G, CRF02_AG | viral RNA | 2532-2549 | reverse | AAAATTTAAAGTACAACC |
| OBV29 | cDNA synthesis subtypes A, CRF01_AE | subtypes A, CRF01_AE | viral RNA | 2532-2549 | reverse | GAAATTTAAAGTACAACC |
| OBV30 | cDNA synthesis subtype C | subtype C | viral RNA | 2532-2549 | reverse | AAAATTTAGTGTGCATCC |
| OBV36 | cDNA synthesis subtype O | group O | viral RNA | 2532-2549 | reverse | GAAATTTAGTGTACAACC |
| p24 | cDNA synthesis | before first round PCR | viral RNA | 3558-3583 | reverse | GGCTCTTGATAAATTTGATATGTCCA |
| MSF12 | forward PCR | first round PCR | cDNA | 623-649 | forward | AAATCTCTAGCAGTGGCGCCCGAACAG |
| PR-R1 | reverse PCR | first round PCR | cDNA | 2591-2611 | reverse | ACTTTTGGGCCATCCATTCCT |
| OBV17 | N-terminus gag-pr amplification; MluI overhang | all subtypes | cDNA | 790-807 (+5' overhang) | forward | GGCGACGCGTGAGAGATGGGTGCGAGAGCGTCA |
| OBV37 | N-terminus gag-pr amplification; MluI overhang | group O | cDNA | 790-807 (+5' overhang) | forward | GGCGACGCGTGAGAGATGGGTGCGAGAGCGTCT |
| OBV18 | C-terminus gag-pr amplification; PvuI overhang | all subtypes | cDNA | 2523-2549 (+3' overhang) | reverse | GTCTCGATCGGACTAATGGGAAAATTTAAAGTGCAGCCAATCTGAGT |
| OBV33 | C-terminus gag-pr amplification; PvuI overhang | subtype A | cDNA | 2523-2549 (+3' overhang) | reverse | GTCTCGATCGGACTAATGGGGAAATTTAAAGTACAACCAATCTGGGT |
| OBV31 | C-terminus gag-pr amplification; PvuI overhang | subtypes B, D, F1 | cDNA | 2523-2549 (+3' overhang) | reverse | GTCTCGATCGGACTAATGGGAAAATTTAAAGTGCAACCAATCTGAGT |
| OBV19 | C-terminus gag-pr amplification; PvuI overhang | subtype C | cDNA | 2523-2549 (+3' overhang) | reverse | GTCTCGATCGGACTAATGGGAAAATTTAAAGTGCAGCCAAGCTGAGT |
| OBV34 | C-terminus gag-pr amplification; PvuI overhang | subtype C | cDNA | 2523-2549 (+3' overhang) | reverse | GTCTCGATCGGACTAATGGGAAAATTTAGTGTGCATCCAAGCTGAGT |
| OBV32 | C-terminus gag-pr amplification; PvuI overhang | subtypes G, CRF02_AG | cDNA | 2523-2549 (+3' overhang) | reverse | GTCTCGATCGGACTAATGGGAAAATTTAAAGTACAACCAATCTGAGT |
| OBV35 | C-terminus gag-pr amplification; PvuI overhang | subtype CRF01_AE | cDNA | 2523-2549 (+3' overhang) | reverse | GTCTCGATCGGACTAATGGGGAAATTTAAAGTACAACCAATCTGAGT |
| OBV38 | C-terminus gag-pr amplification; PvuI overhang | group O | cDNA | 2523-2549 (+3' overhang) | reverse | GTCTCGATCGGACTAATGGGGAAATTTAGTGTACAACCTAATCCTGT |
| OBV21 | sequencing cloned gag-pr | all subtypes | VLP plasmids | 780-801* | forward | GTAATAAGCTTCGAGGTCCGCG |
| OBV22 | sequencing cloned gag-pr | all subtypes | VLP plasmids | 1372-1394* | forward | CAGCATTATCAGAAGGAGCCACC |
| OBV23 | sequencing cloned gag-pr | all subtypes | VLP plasmids | 2059-2079* | forward | CCAAAAATTGCAGGGCCCCTA |
| OBV24 | sequencing cloned gag-pr | all subtypes | VLP plasmids | 2657-2681* | reverse | GTTTAACTTTTGGGCCATCCATTCC |
| OBV25 | sequencing cloned gag-pr | all subtypes | VLP plasmids | 2059-2079* | reverse | TAGGGGCCCTGCAATTTTTGG |
| OBV26 | sequencing cloned gag-pr | all subtypes | VLP plasmids | 1503-1523* | reverse | AATAGGCCCTGCATGCACTGG |
| OBV76 | sequencing cloned gag-pr | group O | VLP plasmids | 1372-1394* | forward | GCATGGGTAAAGGCAGTAGAAG |
| OBV77 | sequencing cloned gag-pr | group O | VLP plasmids | 2059-2079* | forward | GAGGACCAACTCATAAGGCCAGAG |
| OBV78 | sequencing cloned gag-pr | group O | VLP plasmids | 2059-2079* | reverse | GGTGACCTTCCTGTCCACATTTC |
| OBV79 | sequencing cloned gag-pr | group O | VLP plasmids | 1503-1523* | reverse | CTACTGCTTCCTCATTGATTAC |
| Subtype specific primers were generated by downloading sequences from the LANL database to derive a consensus sequence. | | | | |  |  |
| *position in pCMVΔ8.91, all others in HXB2 K03455 reference genome | | |  |  |  |  |

| Table S2: PCR cycling conditions | | | | | |  |  |  |  |  |
| --- | --- | --- | --- | --- | --- | --- | --- | --- | --- | --- |
| ***gag-pr* PCR**  **from cDNA** | | |  | **first round PCR**  **from cDNA** | | |  | **nested *gag-pr* PCR** | | |
|  | 95°C | 5 min |  |  | 94°C | 1 min |  |  | 98°C | 30 secs |
| 45 cycles | 95°C | 15 secs |  | 2 cycles | 94°C | 15 secs |  | 20 cycles | 98°C | 5 secs |
|  | 60°C | 30 secs |  |  | 55°C | 30 secs |  |  | 60°C | 10 secs |
|  | 68°C | 4 min |  |  | 72°C | 3 min |  |  | 72°C | 45 secs |
|  | 68°C | 5 min |  | 3 cycles | 94°C | 15 secs |  |  | 72°C | 60 secs |
|  |  |  |  |  | 57.5°C | 30 secs |  |  |  |  |
|  |  |  |  |  | 72°C | 3 min |  |  |  |  |
|  |  |  |  | 15 cycles | 94°C | 15 secs |  |  |  |  |
|  |  |  |  |  | 60°C | 30 secs |  |  |  |  |
|  |  |  |  |  | 72°C | 3 min |  |  |  |  |
|  |  |  |  |  | 72°C | 60 secs |  |  |  |  |

| Table S3: NCBI GenBank accession numbers for *gag-pr* VLP nucleotide sequences | |
| --- | --- |
| **VLP name** | **accession number** |
| pBV20-A1 | KJ689259 |
| pBV22-A1 | KJ689261 |
| pBV23-A1 | KJ689262 |
| pBV26-A1 | KJ689264 |
| pBV48-A1 | KJ689279 |
| pBV8-B | KJ689249 |
| pBV11-B | KJ689251 |
| pBV15-B | KJ689255 |
| pBV21-B | KJ689260 |
| pBV25-B | KJ689263 |
| pBV34-B | KJ689271 |
| pBV14-C | KJ689254 |
| pBV27-C | KJ689265 |
| pBV31-C | KJ689269 |
| pBV35-C | KJ689272 |
| pBV19-C | KJ689258 |
| pBV38-D | KJ689274 |
| pBV63-D | KJ689289 |
| pBV43-D | KJ689277 |
| pBV60-D | KJ689287 |
| pBV46-BFrec | KJ689278 |
| pBV55-12BF | KJ689283 |
| pBV29-12BF | KJ689267 |
| pBV42-12BF | KJ689276 |
| pBV59-F2 | KJ689286 |
| pBV53-G | KJ689282 |
| pBV61-G | KJ689288 |
| pBV37-G/43_02G | KJ689273 |
| pBV52-G/43_02G | KJ689281 |
| pBV30-20BG | KJ689268 |
| pBV28-01AE | KJ689266 |
| pBV56-01AE | KJ689284 |
| pBV12-01AE | KJ689252 |
| pBV18-01AE | KJ689257 |
| pBV10-02AG | KJ689250 |
| pBV13-02AG | KJ689253 |
| pBV17-02AG | KJ689256 |
| pBV32-02AG | KJ689270 |
| pBV58-02AG | KJ689285 |
| pBV64-H | KJ689290 |
| pBV39-O | KJ689275 |
| pBV51-O | KJ689280 |

| Table S4: p24 quantification of WHO standard on the bioMérieux VIDAS p24 II | | | |
| --- | --- | --- | --- |
| **input** | **measured** |  |  |
| **WHO IU/ml** | **p24 pg/ml** |  |  |
| 20.0 | 105.5 |  |  |
| 10.0 | 56.2 |  |  |
| 4.0 | 21.6 |  |  |
| 1.0 | 4.9 |  |  |
| 0.8 | 3.6 |  |  |

|  | Table S5: Commercial HIV antigen/antibody and antigen-only tests evaluated in this study | | | |  |  |  |  |
| --- | --- | --- | --- | --- | --- | --- | --- | --- |
|  | **manufacturer** | **tradename** | **analysis platform** | **format** | **use**^a^ | **readout**^a^ | **p24 LOD stated by manufacturer** | **laboratory** |
| 4th generation combo | Abbott | ARCHITECT HIV Ag/Ab Combo | Architect | CMIA | Qualitative | S/Co | 4.9 pg/ml^b^ | SNCR, University of Zürich |
|  | Abbott | Prism HIV Ag/Ab Combo | Prism | ChLIA | Qualitative | S/Co | 9.2 pg/ml^b^ | DRK, Hagen |
|  | bioMérieux | VIDAS HIV DuoUltra | VIDAS | ELFA | Qualitative | S/Co | 0.5 IU/ml | University of Bern and Synlab Lucerne^d^ |
|  | BioRad | Access HIV combo | Access | CMIA | Qualitative | S/Co | 1.1 IU/ml | SNCR, University of Zürich |
|  | BioRad | Genscreen Ultra HIV Ag-Ab | microplate | ELISA | Qualitative | S/Co | 0.85 IU/ml | ITG, Antwerp |
|  | DiaSorin | Murex HIV Ag/Ab Combination | microplate | ELISA | Qualitative | S/Co | 7.7 pg/ml | ZLMSG, St.Gallen |
|  | Roche | Elecsys HIV combi PT | COBAS e411 | ECLIA | Qualitative | S/Co | 2 IU/ml | HUG, Geneva |
|  | Siemens | HIV Ag/Ab Combo (CHIV) | ADVIA Centaur | CMIA | Qualitative | S/Co | 1.15 IU/ml | Viollier AG, Allschwil |
|  | Siemens | Enzygnost HIV Integral II | microplate, Quadriga BeFree | ELISA | Qualitative | S/Co | 0.71 IU/ml (typically <1 IU/ml) | BSDSRK, Bern |
|  | Siemens | Enzygnost HIV Integral 4 | microplate, Quadriga BeFree | ELISA | Qualitative | S/Co | 0.29 IU/ml (typically <0.5 IU/ml) | BSDSRK, Bern |
|  | Alere | Determine HIV-1/2 Ag/Ab Combo | manual | LF | Qualitative | reactivity | 2 IU/ml | KSSG, St.Gallen |
|  |  |  |  |  |  |  |  |  |
| antigen-only | bioMérieux | VIDAS HIV p24 II | VIDAS | ELFA | Quantitative | pg/ml | 0.75 IU/ml | University of Basel |
|  | BioRad | Genscreen HIV-1 Ag Assay | microplate | ELISA | Qualitative | S/Co | 0.36 IU/ml | University Hospital, Zürich |
|  | Innogenetics | Innotest HIV Antigen mAb | microplate | ELISA | Qualitative | S/Co | 0.71 IU/ml | ITG, Antwerp |
|  | Perkin Elmer | Alliance HIV p24 antigen ELISA^c^ | microplate | ELISA | Quantitative | pg/ml | 1.8 pg/ml | SNCR, University of Zürich |
|  | Roche | HIV antigen | COBAS MODULAR E170 | ECLIA | Qualitative | S/Co | 0.5 IU/ml | CHUV, Lausanne |
|  | CMIA | chemiluminescent microparticle immunoassay | |  |  |  |  |  |
|  | ChLIA | chemiluminescent immunoassay |  |  |  |  |  |  |
|  | ELFA | enzyme-linked fluorescence assay |  |  |  |  |  |  |
|  | ELISA | enzyme-linked immunosorbant assay |  |  |  |  |  |  |
|  | ECLIA | electro-chemiluminescence immunoassay | |  |  |  |  |  |
|  | LF | lateral flow |  |  |  |  |  |  |
|  | ^a^ as used in this study | |  |  |  |  |  |  |
|  | ^b^ determined using the AFSSAPS panel, which is expressed as an HIV antigen concentration where 1 pg/ml p24 antigen = 3.65 pg/ml HIV antigen [32]. The HIV antigen values provided by the manufacturer were divided by 3.65 to display p24 pg/ml in this table. | | | | | | | |
|  | ^c^ in combination with the Perkin Elmer ELAST ELISA Amplification System  ^d^ University Bern analysed the full panel, Synlab Lucerne analysed heat-denatured samples | | |  |  |  |  |  |
|  | IU/ml = international units/ml of the WHO p24 standard (NIBSC) | | |  |  |  |  |  |
